# Factors influencing COVID-19 health protective behaviours in Zambian university students with symptoms of low mood

**DOI:** 10.1186/s12889-023-15073-4

**Published:** 2023-02-15

**Authors:** Joyce M. Ncheka, J. Anitha Menon, E Bethan Davies, Ravi Paul, Sydney O. C. Mwaba, John Mudenda, Heather Wharrad, Parisa Toutounchi, Cris Glazebrook

**Affiliations:** 1 Department of Psychiatry, School of Medicine, University of Zambia, Lusaka, Zambia; 2 Department of Psychology, School of Humanities and Social Sciences, University of Zambia, Lusaka, Zambia; 3 Department of International Studies, American University of Sharjah, Sharjah, UAE; 4 Institute of Mental Health, NIHR MindTech MedTech Co-Operative, The University of Nottingham, Nottingham, UK; 5 Clinical Neurosciences and Mental Health, School of Medicine, The University of Nottingham, Nottingham, UK; 6 Lusaka Apex Medical University, Lusaka, Zambia; 7 School of Health Sciences, University of Nottingham, Nottingham, UK

**Keywords:** Mental health, COVID-19 protective behaviours, Self-efficacy, Gender

## Abstract

**Background:**

Health protective behaviours are crucial in the prevention of the spread of COVID-19, particularly in university students who typically live and study in large groups. Depression and anxiety are common in students and can impact young people’s motivations to follow health advice. The study aims to assess the relationship between mental health and COVID-19 health-protective behaviours in Zambian university students with symptoms of low mood.

**Methods:**

The study was a cross-sectional, online survey of Zambian university students. Participants were also invited to take part in a semi-structured interview to explore views about COVID-19 vaccination. Invitation emails were sent explaining the study aims and directed students who self-identified as having low mood in the past two weeks to an online survey. Measures included COVID-19 preventive behaviours, COVID-19-related self-efficacy, and Hospital and Anxiety Depression scale.

**Results:**

A total of 620 students (*n*=308 female, *n*=306 male) participated in the study, with a mean participant age of 22.47±3.29 years (range 18–51). Students reported a mean protective behaviour score of 74.09/105 and 74% scored above the threshold for possible anxiety disorder. Three-way ANOVA showed lower COVID-19 protective behaviours in students with possible anxiety disorder (*p*=.024) and those with low self-efficacy (*p*<0.001). Only 168 (27%) said they would accept vaccination against COVID-19, with male students being twice as likely to be willing to accept COVID-19 vaccination (*p*<0.001). Of 50 students interviewed. 30 (60%) expressed fears about the vaccination and 16 (32%) were concerned about a lack of information. Only 8 (16%) participants expressed doubts about effectiveness.

**Conclusion:**

Students who self-identify as having symptoms of depression have high levels of anxiety. The results suggest that interventions to reduce anxiety and promote self-efficacy might enhance students’ COVID-19 protective behaviours. Qualitative data provided insight into the high rates of vaccine hesitancy in this population.

## Background

The first two cases of SARS-CoV-2 (COVID-19) in Zambia were recorded on 18 March 2020 [1]. Since then, despite a relatively low reported death toll, the secondary effects of the pandemic have stretched social and economic sectors, and rural healthcare systems have been pushed beyond capacity [2]. A systematic review of excess deaths globally suggests that the impact of the COVID-19 pandemic on mortality rates is likely to have been more devastating in sub-Saharan Africa than that indicated by the official statistics for COVID-19 deaths. [3]. For university students, additional disruptions to academic life such as lack of access to Information and Communications Technology (ICT) off-campus prevent participation in e-learning classes, thus causing a widening socio-economic divide in higher education and jeopardising students’ academic achievements and future careers [4].

Although seen as disruptive by students, health-protective behaviours such as mask-wearing, social distancing, and hand washing are crucial to preventing the spread of COVID-19: particularly in university settings where young people live and study in large groups. However, COVID-19 restrictions may be challenging for university students at a time when they are forging new social relationships and subject to intense peer pressure. A recent cross-sectional survey of Ethiopian students found that, although 70% of students were willing to wear a mask, the proportion of students with high levels of preparedness was low (19%), with male students being less prepared than female students. Furthermore, only half of the sample scored highly for self-efficacy, the belief in one’s ability to implement a particular behaviour, which is a strong determinant of health-protective behaviours [5].

To implement effective health-protective strategies, it is important to understand barriers and facilitators for behaviour change and pandemics can provide an important context for such research. A review of 26 studies of health-protective behaviours associated with previous pandemics, including SARS and swine flu, explored social cognition and demographic factors influencing compliance with recommended behavioural adaptations [6]. Factors that emerged consistently across some studies as increasing rates of health-protective behaviours or intentions to adhere to prevention strategies included female gender, older age, higher educational level, increased perceived susceptibility to the pandemic disease, and higher levels of self-efficacy.

Although less physically vulnerable to COVID-19, arguably the impact of the pandemic on student mental health may be significant, due to disruption of social support and additional educational pressures. A meta-analysis of studies evaluating the mental health of college students conducted between December 2019 and October 2020 concluded that rates of depression had increased substantially from 21% (95% CI: 16–25%) before March 2020 to 54% (95% CI: 40–67%) between December 2019 and October 2020 [7]. Rates of anxiety had also increased substantially after the start of pandemic restrictions. A longitudinal US study found that rates of moderate to severe depression in students increased from 10% to 31.7% in the four months following the start of the pandemic with women being more affected than men [8]. Students in low-income countries may be particularly affected by the constraints of the pandemic due to limited access to remote learning and greater economic vulnerability. A survey of South African undergraduate students conducted during the pandemic reported high levels of psychological distress and found that higher levels of hopelessness and anxiety were associated with lower levels of health-protective behaviours [9]. This may reflect the strong association between poor mental health and low self-efficacy [10].

The COVID-19 pandemic offers an opportunity to explore the impact of poor mental health on facilitators of healthy behaviour such as self-efficacy, and also directly on health-protective behaviours themselves. These relationships are likely to be particularly significant for less resilient students. Therefore, this study recruited a large sample of students with self-reported low mood during the peak of the COVID-19 outbreak in Zambia. The findings will inform our understanding of the health promotion strategies needed to protect vulnerable students during a pandemic. The study is underpinned by social cognition theories (Theory of Planned Behaviour and the Revised Health Model) which argue that health behaviour is driven by beliefs about outcomes of the behaviour and motivation and feelings of capability regarding the ability to carry out the behaviour. Objectives are to determine rates of health-protective behaviours in students with symptoms of low mood and to explore the association between mental health and self-efficacy and health-protective behaviours in vulnerable students. A further objective is to explore rates of vaccine hesitancy and to understand factors influencing up take of COVID-19 vaccination. We hypothesize that students with low self-efficacy will report fewer health protective behaviours.

## Methods

### Design

This was a cross-sectional online survey, and the participants were Zambian university students at risk for depression. Data were collected between February-July 2021 as baseline assessment for a longitudinal, mixed-methods feasibility study of an online cognitive behavioural therapy (CBT) based online intervention to promote emotional and behavioural resilience during the COVID-19 pandemic. Participants were also given the opportunity to take part in a semi-structured interview and the responses to a question about COVID-19 vaccination were analysed as part of this study.

### Participants and data collection procedures

Non-probability sampling was used to recruit students at risk of depression from the University of Zambia (UNZA) and Lusaka Apex Medical University (LAMU). The pragmatic target sample size was 1000 students. To be eligible to participate, university students had to be aged≥18 years, in their second year of study or beyond, and self-identify as having experienced at least one of two key symptoms of depression in the previous two weeks [11]. Due to the longitudinal nature of the intervention, students in their final year of study were not eligible to participate.

To avoid potential bias invitation, emails were sent to all registered students meeting the inclusion criteria for the year of study (*n*=21,450) via UNZA Centre for Information Communication Technologies (CICT) and LAMU information technology department. The invitation email explained the study aims and directed students who self-identified as having low mood in the past two weeks (little interest or pleasure in doing things and/or, feeling down, depressed, or hopeless) to an online survey hosted on JISC Online Surveys [12]. Invitation reminders were emailed two and four weeks after the initial invitation. The anonymous survey included a detailed participant information sheet and a tick-box consent form and took approximately 20 min to complete. It was written in English and comprised questions on socio-economic factors, health-protective behaviours, beliefs and attitudes towards COVID-19 and academic performance (not reported in this study). Fifty students who agreed to a recorded telephone/ online interview were contacted by email to arrange a suitable time.

### Measures

Participants reported socio-demographic details including age, gender, year of study, university major and parental education. An adapted version of the Material Affluence Scale (MAS) [13] was used to assess family economic status. This 7-item scale was devised specifically for use in low-income countries and probes economic indicators associated with the family home such as car ownership, electricity and type of construction of the home. Total MAS scores range from 0 to 7, with a higher MAS score indicating higher family economic status. Total MAS scores are a valid and easily collected measure of family economic status, correlating with parental social economic status, parental education and parental occupation. Cronbach’s alpha was acceptable (0.62) for the 9-item version which included 2 items with very low item total correlations which were not included in the present study [13].

#### Preventative behaviours

The Preventive Behaviours Scale (PBS) was developed for the study based on WHO guidelines [14] and adapted from a questionnaire developed for the influenza H1N1 pandemic [15]. The 16 items rated the frequency of recommended COVID-19 preventive behaviours in the past seven days such as hand washing, isolation, vaccination and wearing face masks, on a 1 to 7 scale from ‘not at all’ to ‘very much so’. Total scores (excluding the item rating self-isolation if tested positive) ranged from 15–105, with higher scores indicating stronger compliance with COVID-19 prevention behaviours.

#### Self-efficacy

To assess perceived self-efficacy to protect oneself against COVID-19, a valid two-item scale [16] again adapted from WHO guidelines [14] was used to assess confidence in the ability to protect oneself against COVID-19. The items are measured on a scale from 1 to 7, with total scores ranging from 2 to 14, and higher scores indicating a higher level of self-efficacy and preparedness to carry out COVID-19 protective behaviours. Participants scoring below the median (2 to 10) were allocated to the low self-efficacy group and participants scoring above the median (≥11) were allocated to the high self-efficacy group.

#### Vaccination uptake

A single item rated ‘Yes’, ‘No’ or ‘Unsure’ was used to assess the willingness to take up a potential offer of a COVID-19 vaccination. To create a binary variable, ‘No’ or ‘Unsure’ responses were recoded as 0 (not accept), and ‘Yes’ responses recoded as 1 (accept).

#### Mental health

The Hospital Anxiety and Depression Scale (HADS) assessed students’ depressive and anxiety symptomology [17]. It consists of two seven-item subscales reflecting depressive and anxiety symptomology respectively, with higher scores indicating greater symptomology A threshold score of≥8 was used on each subscale to classify participants as experiencing possible clinically significant anxiety or depression [18]. The HADS has been used previously with South African students to determine rates of depression and anxiety among medical students and to examine the associations with various socio-demographic variables [19]. One review found that across 747 papers that used HADS, the mean Cronbach’s alpha for the anxiety subscale was α=0.83 (range 0.68 to 0.93), and α=0.82 for the depression scale (range of 0.67 to 0.90) [18].

The interview proforma included an open question probing views about vaccination (How do you feel about vaccination against COVID-19?).

### Ethical approval

Ethical approval was obtained from UNZA Biomedical Research ethics committee (UNZABREC), the National Health Research Authority (NHRA) and permissions were sought from the management of the University of Zambia and Lusaka Apex Medical University. Participants were required to complete an online, tick box informed consent form to participate in the anonymous survey and the follow-up interview.

### Data analysis

Data were exported and analysed using SPSS version 27 [20]. Pearson correlations were conducted to explore relationships between PBS scores and demographic and mental health. A three-way Analysis of Variance (ANOVA) was conducted with mental health (depression/no-depression), self-efficacy (high/low), and gender as the independent variables and the total Protective Behaviour Scale score as the dependent variable. Chi-square was used to examine the relationship between vaccine willingness and depression and anxiety groups, and independent t-tests to examine group differences. Participants with missing values for the HADS were excluded but no other adjustments for missing values were required. Content analysis was used to analyse responses to the question about views on the COVID-19 vaccination.

## Results

A total of 633 participants completed the online survey. One participant was excluded from. the database for not meeting the inclusion criteria, and a further 12 participants (4 females, 8 male) were removed from the data set due to missing data values for the HADS. The remaining 620 participants (308 females, 306 males, 6 unspecified) had a mean age of 22.47 years (SD=3.29; range 18–51). Table 1 shows the full demographic information for the sample.

**Table 1:** Socio-demographic characteristics of the sample

Gender	
Male	306 (49.40%)
Female	308 (49.70%)
Other	1 (0.20%)
Prefer not to say	5 (0.80%)
Age	
18 – 19	39 (6.30%)
20 – 29	511 (82.40%)
30 +	17 (3.00%)
Prefer not to say	53 (8.50%)
Mean age (SD)	22.47 (SD 29.00)
Marital status	
Single	596 (96.10%)
Married	17 (2.70%)
Divorced	1 (0.20%)
Prefer not to say	6 (1.00%)
Year of study	
Second year	256 (41.30%)
Third year	217 (35.00%)
Fourth year	83 (13.40%)
Sixth year	29 (4.70%)
Prefer not to say	35 (5.60%)
Department of study	
Agriculture UNZA	19 (3.10%)
Engineering UNZA	50 (8.10%)
Education UNZA	103 (16.60%)
Humanities and social sciences UNZA	90 (14.50%)
Law UNZA	55 (8.90%)
Mines UNZA	5 (0.80%)
Medicine UNZA	129 (20.80%)
Natural sciences UNZA	64 (10.30%)
Veterinary medicine UNZA	9 (1.50%)
Medicine LAMU	81 (13.10%)
Prefer not to say	15 (2.40%)
Mean Material Affluence Scale score (SD)	9.0 (SD 1.79)

Internal consistency was acceptable for the HADS depression subscale (α=0.68) and good for the HADS anxiety subscale (α=0.81) and the Protective Behaviours Scale (α=0.89).

The two-item self-efficacy scale had only moderate internal consistency (α=0.51). The study found that 335 students (54%) scored above the threshold for possible depressive disorder and 460 students (74.2%) scored above the threshold for possible anxiety disorder. Females had significantly higher scores for depression (t=2.01, df=612, *p*=0.045) and anxiety (t=-3.27, df=612, *p*<0.001). Compared to males, a higher proportion of females scored above the cutoff for anxiety (X^2^=11.27, df=1, *p*<0.001) and above the cutoff for depression X^2^=5.48, df=1, *p*=0.019). There was no difference in self-efficacy between males and females (*p*=0.05) (Table 2).

**Table 2:** Mental health outcomes and self-efficacy by gender

	**Male (*n* = 306)**	**Female (*n* = 308)**	**Total (*n* = 620)**	***P* value**
Mean HADS-D (SD)	7.6 (SD 3.64)	8.26 (3.71)	7.99 (3.67)	0.045
Case depression (%)	150 (49.00%)	180 (58.40%)	335 (54%)	0.019
Mean HADS-A (SD)	10.18 (SD 4.60)	11.36 (SD 4.31)	10.81 (4.52)	< 0.001
Case anxiety (%)	208 (68.00%)	246 (79.90%)	460 (74.2%)	< 0.001
Mean self-efficacy (SD)	11.00 (2.45)	11.02 (2.32)	11.00 (2.34)	ns

*ns* not significant

### Factors influencing health-protective behaviours

Students reported a mean protective behaviour score (PBS) of 74.09 (out of 105). Just over a quarter (*n*=168, 27.00%) said they would accept vaccination against COVID-19, *n*=333 (54.20%) were unsure and *n*=113 (18.40%) would refuse a vaccination if they were offered one. There was no significant difference in total PBS scores between males and females (*p*=>0.05) but over a third of males (*n*=105, 34.30%) reported they would accept a vaccination, compared to only a fifth (*n*=63, 20.50%) of female students (X^2^=14.83, d=1, *P*<0.001; OR 2.03, CIs 1.41- 2.92). Thus, males were twice as likely to agree that they would accept a COVID-19 vaccination if they were offered one.

Higher reported total PBS scores were associated with higher self-efficacy (*r*=0.24, *n*=620, *p*<0.001) and lower levels of anxiety (*r*=-0.103, *n*=620, *p*=0.01). Age, family affluence and depression were not significantly related to PBS (all *p*=>0.05). Both depression (*r*=-0.106, *p*<0.001) and anxiety (*r*=-0.1, *p*=0.013) were negatively associated with perceptions of self-efficacy for protecting against COVID-19. Participants scoring above the cut-off for possible clinically significant anxiety had lower PBS scores (t=2.4, df=618, *p*=0.017) and lower self-efficacy scores (t=2.25, df=218, *p*=0.025). Participants scoring above the cut-off for possible clinically significant depression disorder had lower self-efficacy scores (t=2.29 df=218, *p*=0.02) but not lower PBS scores (Table 3).

**Table 3:** PBS scores and self-efficacy by anxiety caseness

	**Possible case of clinically significant anxiety** (*n* = 460)	**Non-case of clinically significant anxiety** (*n* = 160)	***P* value**
Mean PBS (SD)	72.95 (19.98)	77.33 (19.65)	0.017
Mean self-efficacy (SD)	10.87 (2.32)	11.36 (2.37)	0.025

A three-way ANOVA found main effects for anxiety group (F=5.00, df=1,618, *p*=0.024, eta squared=0.008) and self-efficacy group (F=13.1, df=1,618, *p*<0.001, eta squared=0.021). Students scoring above the cut-off for possible clinically significant anxiety and students with low-self efficacy had lower self-reported total COVID-19 protective behaviour scores (Table 3). There were no significant interactions between the variables. There was no relationship between willingness to be vaccinated and either anxiety or depression. Mean scores for individual COVID-19 protective behaviours are shown in Table 4. The anxiety group had lower scores for mask-wearing in class, mask-wearing outside when socially distancing was not possible, and mask-wearing when visiting family and friends. They also had lower scores for keeping social distance in class and keeping social distance outside but higher scores for isolating if they suspected they had COVID-19 symptoms.

**Table 4:** Health protective behaviours (*n*=620) and differences in mental health status

	**Mean behaviour (SD)**	**Never carried out behaviour (%)**	**Case anxiety vs Non-anxiety Case**
Frequently washed my hands with soap and water	5.43 (1.76)	31 (5.00%)	ns
Avoided touching my eyes, nose and mouth with unwashed hands	4.45 (1.99)	73 (11.80%)	ns
Used disinfectants to clean hands when soap and water were not available	2.104 (5.19)	76 (12.30%)	ns
Avoided mixing with large groups of friends	4.94 (2.10)	72 (11.60%)	ns
Wore a mask during classes	5.27 (2.39)	126 (20.30%)	t = 2.11, *p* = 0.03
Wore a mask on the bus	5.77 (2.0)	67 (10.80%)	ns
Wore a mask in shops	5.96 (1.83)	49 (7.90%)	ns
Wore a mask outside when I couldn’t socially distance	5.30 (2.12)	80 (12.90%)	t = 2.42, *p* = 0.016
Wore a mask to visit family or friends	4.39 (2.19)	114 (18.40%)	t = 3.23, *p* = 0.001
Kept my distance with a group of friends outside	4.33 (2.14)	100 (16.10%)	t = 2.91, *p* = 0.004
Kept my distance with a group of friends inside	3.90 (2.17)	135 (21.80%)	ns
Isolated myself when I thought I might have COVID-19 symptoms	3.99 (2.58)	226 (36.50%)	t = -2.57, *p* = 0.01 (Anx > Non-Anx)
Wore a mask outside in a large group	5.53 (2.10)	79 (12.70%)	ns
Kept my distance in shops	5.39 (2.00)	67 (10.800%)	ns
Kept my distance in class	4.25 (2.43)	167 (26.90%)	t = 2.04, *p* = 0.041
Isolated myself when I had a positive COVID-19 test ^a^	4.73 (2.56)	51 (26.40%)	ns

*ns* not significant

^a^*n* = 193 (Not included in PBS total)

### Content analysis of views about vaccination

Fifty participants were interviewed (26 males and 24 females). Thirty (60%) participants (15 males and 15 females) expressed fears about the vaccination and its side effects. A common anxiety was that the vaccination programme was part of a plot to reduce the population.

“Some are saying they are injecting chips. I don’t know, to control us here in Africa, to kill us” (P 38).

Linked to this were concerns that the vaccination had magnetic properties.

"I just saw on social media, the injection site leaves a magnetic effect. Now the site can switch on a bulb without any connection” (P 30).

These views seemed to be reinforced by the perception that Africa had been sent vaccinations not used in higher income countries.

"Why is it labelled for export only? Is it just for us… the Africans? We need answers to these questions?” (P25).

Nearly a third (32%) expressed a need for more information about the vaccine (9 males, 7 females).

..”people are hearing a lot on social media. If the people giving can say something… we will listen to then, we have no other choice. The people giving vaccinations are not giving out any information, they need to say how the vaccine will help” (P20).

But only 8 (16%) participants voiced doubts about the effectiveness of the vaccination.

## Discussion

In this group of students who identified as having at least one symptom of low mood during the COVID-19 pandemic, over half (54.00%) scored above the cut-off for possible depression. There were very high rates of anxiety (74.00%) within this vulnerable sample, with females having higher scores for both anxiety and depression. Levels of self-reported protective behaviours were generally high but a fifth of students would not wear a mask in class and a quarter did not socially distance themselves in class. There was evidence of high levels of vaccine hesitancy with only 27.00% of students reporting they would accept vaccination; with the majority reporting, they were unsure.

Higher levels of depression and anxiety were associated with lower self-efficacy for protecting against COVID-19 but only anxiety was associated with poorer COVID-19 protective behaviours. ANOVA revealed independent main effects for low self-efficacy and possible anxiety disorder on lower PBS scores but no effect of gender and no significant interactions.

A systematic review of 36 studies evaluating the prevalence of anxiety disorders in university students during the COVID-19 pandemic reported rates ranging between 11.00% and 89.00%, with an average prevalence of 41.00% (95% CI=0.34–0.49) [21]. The majority of studies were carried out in Asia, with only one from Africa (Egypt). The results of our study suggest that Zambian students who identify as having symptoms of low mood have relatively high rates of possible anxiety disorder. However, a study of Bangladeshi students also found high rates of anxiety with 87.70% of the 392 students recruited above the threshold for mild to severe anxiety [22].

The high rate of vaccine hesitancy in this student sample (73.00%) is in contrast to studies of student populations in Lebanon (13.00%) [23], Italy (13.90%) [24], the US (46.00%) [25] and Ethiopia (41.20%) [26]. Concerns about safety and lack of awareness of effectiveness appear to be driving the high rates of vaccine hesitancy in Zambia [27, 28]. Although official figures appear to suggest a low death rate in Zambia from COVID-19, addressing vaccine hesitancy is vital as morgue data and excess death figures paint a much bleaker picture [3, 29]. Our qualitative data confirmed high levels of fear associated with the COVID-19 vaccine, even in this educated sample, and suggested that social media had fueled those fears in the absence of information from reliable sources. Only a small proportion of our sub-sample expressed concerns about vaccine effectiveness suggesting that information should also focus on safety and health care providers’ motivation for providing treatment. A recent study with Nigerian victims of insurgency demonstrated that pictorial information about COVID-19 vaccination was associated with greater self-efficacy and stronger intentions to take up vaccination [30].

In our study, female students had greater vaccine hesitancy which has also been observed in other studies with student populations [25]. A systematic review to explore gender differences in COVID-19 vaccination intentions found males were more likely to be intending to accept a COVID-19 vaccine in 35/60 studies (58.00%). Meta-analysis of data from 46 of the included studies found the odds of men accepting vaccination were 1.4 times higher than women. (95% CI 1.28–1.55) [31]. In our study men had twice the acceptance rate compared to women. Our qualitative data failed to illuminate the reasons for gender differences in vaccine hesitancy, suggesting a common pattern of fears and concerns. However, since women have been shown to be less individualistic than men in terms of their decision making and more risk-averse, the same fears and uncertainties may have a differential impact for women when they come to make a judgement about whether to accept COVID-19 vaccination [32, 33].

We found no significant difference in total protective behaviour scores between males and females and overall scores suggested reasonable compliance to recommended protective behaviours. However, some behaviours had relatively high rates of non-compliance with over a fifth of students reporting that they never wore masks in class or socially distanced from friends indoors and over a third reported that they did not socially isolate if they had COVID-19 symptoms. Anxiety was negatively associated with protective behaviours and students scoring above the threshold for possible anxiety disorder had lower rates of total protective behaviours. Students in the high anxiety group were significantly less likely to wear a mask in class, when visiting family or outside when they couldn’t socially distance themselves.

They were also less likely to socially distance themselves in class or outside but were more likely to socially isolate themselves if they suspected symptoms. Relatively few studies have explored the impact of student mental health on COVID-19 protective behaviours but some studies have provided support for the relationship between high anxiety and poorer adherence to health advice. For example, a study in Western India reported an inverse correlation between anxiety and health-protective behaviours in nursing students [34]. However, analysis of data from the US and Australia suggested that anxiety may have functional benefits by mediating the association between information consumption and improved COVID-19 protective behaviours [35]. This supports the role of effective health education to help channel students’ anxiety and build self-efficacy.

We found high self-efficacy was the strongest independent predictor of better health-protective behaviours and both depression and anxiety were inversely related to students’ perceptions of their ability to manage their COVID-19 risk. A study in China found that students with high self-efficacy used more active coping strategies and had better mental health [36], and a study of pregnant women in China also found self-efficacy to be protective against anxiety and depression during the COVID-19 pandemic [37]. Since self-efficacy has been found to mediate the relationship between knowledge of dengue fever and dengue protective behaviour during a dengue fever outbreak in Malaysia [38], it is possible that promoting self-efficacy through effective education could improve health protective behaviours through its impact on self-efficacy and mental health [39].

In recognition of the need for appropriate, culturally tailored Covid-19 education for students delivered through accessible channels such as mobile technology [40], the research team has co-created with students from Africa and other stakeholders, a freely available, multimedia educational package titled ‘Covid-19 Education for African Students (COVEDAS)’ to educate and reassure students about how to keep them and their communities safe from Covid-19 [41]. Sections on COVID-19 vaccination have been informed by the qualitative data from this study and aim to address common myths and concerns. Reducing anxiety may help to increase responsiveness to effective and culturally sensitive health education, thus building self-efficacy for health-protective behaviours, with consequent public health benefits.

The strengths of the current study are that it had a large sample of students (*N*=620) from a low-income country and one which is underrepresented in COVID-19 research. An additional strength is the high proportion of males recruited since they are also under-represented in this type of study. We excluded only 12 students with missing values for the HADS and the remaining sample had complete data for health protective behaviours. A limitation of the study is that we cannot estimate the response rate as only students who self-identified as having symptoms of depression were eligible to participate. Furthermore, recruitment was disrupted by COVID-19 lockdowns with many students returning to their family homes, often with limited internet access. The cross-sectional nature of this study limited our ability to identify causal relationships. However, to our knowledge, this is the first research to explore the potential of mental health to impact COVID-19 prevention behaviours in an African student sample and the first to use a mixed-methods approach to understanding vaccine hesitancy. We demonstrated that participants with poorer mental health perceived themselves as less able to protect themselves from COVID-19 and that more anxious students practised fewer protective health behaviours. Future research should consider replicating this research to a wider African population, including students outside of Zambia to see if the results are replicable to the general African student population. Our findings suggest that interventions are needed to support anxious students during times of health threat.

## Conclusion

Students from a low-income country who self-identified as having symptoms of depression had high levels of anxiety during the COVID-19 pandemic. The results of the study suggest that interventions to reduce anxiety and promote self-efficacy might enhance students’ COVID-19 protective behaviours. It is possible that promoting self-efficacy through effective education could improve health-protective behaviours through its impact on self-efficacy and mental health, but longitudinal research is needed to explore causal relationships in more detail. Qualitative research suggested that high levels of vaccine hesitancy may reflect the influence of poor and misleading information, but more research is needed to understand gender differences in rates of vaccine hesitancy in this population.

## Data Availability

Data are available from the corresponding author on reasonable request.

## Abbreviations

CICT: Centre for Information Communication Technologies
PBS: Preventive Behaviours Score
WHO: World Health Organisation
UNZA: University of Zambia
LAMU: Lusaka Apex Medical University
UNZABREC: University of Zambia Biomedical Research ethics committee
NHRA: National Health Research Authority
OR: Odds Ratio
CIs: Confidence intervals
MAS: Material Affluence Scale
HADS: Hospital Anxiety and Depression Scale
